# The Effectiveness of Photobiomodulation Therapy on Perineal Pain and Wound Healing After Episiotomy—A Systematic Review and Meta-Analysis

**DOI:** 10.3390/jcm15030964

**Published:** 2026-01-25

**Authors:** Mohamed Salaheldien Alayat, Roaa A. Sroge, Fahda M. Alshiakh, Ehab Mohamed Abd El-Kafy, Ammar Fadil, Abdulaziz Awali, Moayad S. Subahi, Abdulqader Abdulrazaq Almutairi

**Affiliations:** 1 Medical Rehabilitation Science Department, College of Applied Medical Science, Umm Al-Qura University, Makkah 21955, Saudi Arabia; rasroge@uqu.edu.sa (R.A.S.); fmshiakh@uqu.edu.sa (F.M.A.); emkafy@uqu.edu.sa (E.M.A.E.-K.); asfadil@uqu.edu.sa (A.F.); amawali@uqu.edu.sa (A.A.); mssubahi@uqu.edu.sa (M.S.S.); 2 Physiotherapy Department, Taif Armed Forces Hospitals, Taif 21944, Saudi Arabia; abdulqader.almutairi@umontana.edu

**Keywords:** episiotomy, photobiomodulation therapy, low-level laser therapy, perineal pain, wound healing, systematic review and meta-analysis

## Abstract

**Objective**: the aim of this systematic review and meta-analysis was to evaluate the effectiveness of photobiomodulation therapy (PBM) on perineal pain and wound healing following episiotomy. **Methods**: Electronic databases were searched for randomized controlled trials (RCTs) and non-RCTs investigating PBM after episiotomy. Risk of bias was assessed using RoB 2 for RCTs and ROBINS-I for non-RCTs. The certainty of evidence was evaluated using the Grading of Recommendations Assessment, Development and Evaluation (GRADE) approach. Meta-analyses were performed using random-effects models. **Results**: Eight studies were included in the systematic review, and six studies were included in the meta-analysis. According to RoB 2, one trial was judged at low risk of bias, two trials raised some concerns, and three trials were at high risk of bias. ROBINS-I showed the serious risk of bias of two non-RCTs. GRADE for both pain and wound-healing outcomes was rated as very low. Meta-analysis of pain showed no significant difference between PBM and control groups (SMD = 0.04; 95% CI −0.60 to 0.68; *p* = 0.89), with considerable heterogeneity (I^2^ = 91%). For wound-healing outcomes, meta-analysis showed no significant difference (MD = 0.94; 95% CI −0.69 to 2.56; *p* = 0.26), with substantial heterogeneity (I^2^ = 94%). **Conclusions**: PBM therapy did not demonstrate a significant benefit for reducing perineal pain or improving wound healing after episiotomy compared with control interventions. Interpretation of these findings should be made cautiously due to small study numbers, substantial heterogeneity, and the inability to perform sensitivity or subgroup analyses, highlighting the need for high-quality RCT with standardized PBM protocols before clinical recommendations can be made.

## 1. Introduction

Episiotomy is a surgical incision of the perineum performed during vaginal delivery to enlarge the vaginal outlet and facilitate childbirth. Although its routine use has declined, episiotomy is still commonly performed in many clinical settings, particularly in primiparous women, instrumental deliveries, shoulder dystocia, and cases of fetal distress [1,2]. Mediolateral episiotomy remains the most frequently used technique due to its lower risk of extension to third- or fourth-degree perineal tears compared with midline episiotomy [3]. Despite evolving guidelines advocating restrictive rather than routine use, episiotomy continues to represent a significant source of postpartum morbidity worldwide [2,4].

Post-episiotomy pain is one of the most common and distressing postpartum complaints, often interfering with sitting, walking, breastfeeding, sleep, and maternal–infant bonding. Pain may persist beyond the immediate postpartum period and, in some cases, evolve into chronic perineal pain or dyspareunia [5,6]. In addition to pain, episiotomy wounds are susceptible to complications such as edema, inflammation, infection, delayed healing, and wound dehiscence [5,6].

Episiotomy-related pain and wound complications represent a substantial burden in low- and middle-income countries, where episiotomy rates often remain high and access to comprehensive postpartum care may be limited [7,8]. In such settings, prolonged perineal pain and delayed wound healing can adversely affect maternal function, caregiving capacity, and return to daily activities, while increasing reliance on pharmacological treatments that may be costly or associated with adverse effects during breastfeeding [7,8]. These complications can prolong recovery, increase healthcare utilization, and negatively impact quality of life during the puerperium [9].

Conventional management of episiotomy-related pain and wound healing includes systemic analgesics, local anesthetics, cryotherapy, sitz baths, topical agents, and antibiotics when infection is suspected [10,11]. While these approaches may provide symptomatic relief, they are associated with variable efficacy and potential adverse effects, particularly with prolonged pharmacological use during breastfeeding [12,13]. Moreover, many conventional interventions focus primarily on pain control rather than actively enhancing tissue repair, highlighting the need for adjunctive, non-pharmacological strategies that can simultaneously address pain and wound healing [10,12,13].

Photobiomodulation therapy (PBM), also referred to as low-level laser therapy (LLLT), involves the application of low-intensity red or near-infrared light to stimulate cellular and biological processes [14]. PBM is thought to exert its effects through absorption of photons by mitochondrial chromophores, particularly cytochrome c oxidase, leading to increased ATP production, modulation of reactive oxygen species, enhanced cell proliferation, angiogenesis, and collagen synthesis [14,15,16]. These mechanisms underpin the potential analgesic, anti-inflammatory, and tissue-repair effects of PBM, making it a promising modality for the management of surgical wounds [16,17].

A growing body of evidence suggests that PBM may accelerate wound healing and reduce pain in various clinical contexts, including chronic ulcers, burns, musculoskeletal injuries, and postoperative wounds [18,19]. Clinical and experimental studies have reported improved epithelialization, reduced inflammation, and enhanced tensile strength of healed tissue following PBM application. Additionally, PBM has demonstrated analgesic effects through modulation of peripheral nerve activity and endogenous opioid pathways [18,20]. However, reported outcomes vary widely across studies, likely due to heterogeneity in PBM parameters, treatment timing, and study design [21].

Several randomized and non-randomized clinical trials have investigated the use of PBM for managing pain and promoting wound healing following episiotomy [22,23,24]. While some studies report favorable effects on pain reduction and faster wound healing, others have found no significant benefit compared with sham or conventional treatments [11,22,25]. The variability in findings raises substantial concerns regarding its clinical effectiveness and leaves its overall impact indeterminate.

Despite increasing interest in PBM as a non-invasive intervention for episiotomy-related pain and wound healing, no consensus has been reached regarding its effectiveness. To date, the available evidence has not been comprehensively synthesized with rigorous assessment of methodological quality, risk of bias, and certainty of evidence. Therefore, a systematic review and meta-analysis aimed to evaluate the effectiveness of PBM therapy on perineal pain and wound healing following episiotomy, compared with sham or conventional control interventions. Specifically, this review sought to synthesize the available evidence, quantify pooled effects on pain and wound-healing outcomes where feasible, and assess the certainty of the evidence using the Grading of Recommendations Assessment, Development and Evaluation (GRADE) framework. Such synthesis is essential to inform clinical decision-making, guide future research, and clarify whether PBM should be recommended as an adjunctive intervention in postpartum care. However, given the limited number of available trials and anticipated clinical and dosimetric heterogeneity, any conclusions drawn from the existing evidence should be made with caution and considered primarily hypothesis-generating rather than definitive.

## 2. Methods

### 2.1. Study Design

This study was conducted as a systematic review and meta-analysis of randomized and non-randomized controlled studies evaluating the effectiveness of PBM therapy on pain and wound healing following episiotomy. The review was designed and reported in accordance with the Preferred Reporting Items for Systematic Reviews and Meta-Analyses (PRISMA) 2020 guidelines [26]. The review protocol was prospectively registered in the International Prospective Register of Systematic Reviews (PROSPERO; registration number CRD420251179085). All scientific content, analyses, and conclusions were conceived, verified, and approved by the authors, who retain full responsibility for the accuracy and integrity of the work. The AI tool did not generate study data, perform statistical analyses, select outcomes, influence methodological decisions, or contribute to data interpretation (Supplementary File S1: PRISMA 2020 checklist).

### 2.2. The Search Strategy

A comprehensive search of the literature was conducted to identify studies evaluating the effectiveness of PBM or laser therapy on pain relief and perineal wound healing in postpartum women following episiotomy or perineal tear repair. The search strategy was initially developed for PubMed (MEDLINE) and subsequently adapted for use in other electronic databases such as Scopus, CINAHL (EBSCOhost), the Cochrane Central Register of Con-125 trolled Trials (CENTRAL), and the Physiotherapy Evidence Database (PEDro). Additional sources, such as Google Scholar and ResearchGate, were screened for gray literature.

The PubMed search was performed from inception to 31 October 2025 by two independent reviewers (R.A.S. and F.M.A.) and was validated by a third reviewer (A.A.). The search was constructed using a combination of Medical Subject Headings (MeSH) and free-text keywords, structured according to the PICO framework. Boolean operators (AND, OR) were applied to combine terms related to the population, intervention, and outcomes. The population component included terms related to the postpartum period, episiotomy, perineal trauma, and vaginal delivery. The intervention component comprised terms describing laser therapy and photobiomodulation, including low-level laser therapy and related synonyms. Outcome terms encompassed pain-related measures and wound-healing indicators, such as inflammation, edema, infection, and scar formation.

PubMed Search terms were ((“Postpartum Period” [Mesh] OR “Puerperium” [Mesh] OR postpartum OR postnatal OR puerperal OR “Episiotomy” [Mesh] OR episiotomy OR “Perineum/injuries” [Mesh] OR “Perineal Lacerations” [Mesh] OR perineal tear OR perineal trauma OR obstetric laceration OR vaginal delivery) AND (“Laser Therapy” [Mesh] OR “Phototherapy” [Mesh] OR “Low-Level Light Therapy” [Mesh] OR “Photobiomodulation Therapy” [Mesh] OR laser therapy OR low-level laser OR low-intensity laser OR diode laser OR infrared laser OR laser irradiation OR photobiomodulation OR LLLT OR PBM) AND (“Pain” [Mesh] OR pain OR perineal pain OR pain relief OR “Wound Healing” [Mesh] OR wound healing OR tissue repair OR healing time OR “Inflammation” [Mesh] OR inflammation OR edema OR infection OR scar OR scar tissue)) (Supplementary File S2: PubMed Search Strategy).

### 2.3. Eligibility Criteria

Studies were eligible for inclusion if they were the following: any clinical trials that evaluated the effect of PBM therapy or low-level laser therapy on pain and/or wound healing after episiotomy. Eligible studies included postpartum women who underwent episiotomy during vaginal delivery. Interventions of interest comprised PBM applied to the episiotomy wound using any wavelength, dose, intensity, duration, frequency, or number of sessions, either alone or as an adjunct to standard postpartum care. Comparator conditions included standard or routine postpartum care, placebo or sham laser therapy, no intervention/usual care, or alternative physical modalities used for episiotomy management. Studies were required to report perineal pain intensity as a primary outcome (assessed using validated or clinically accepted scales) and/or wound healing assessed using validated measures such as the Redness, Edema, Ecchymosis, Discharge, Approximation (REEDA) scale or other clinically relevant healing indicators.

Studies were excluded if they were case reports, case series, narrative reviews, systematic reviews, editorials, letters, conference abstracts, theses, dissertations, or if they involved animal or in vitro experiments. Studies without a comparison group, those not evaluating PBM or laser therapy, or those not conducted in a postpartum episiotomy context were also excluded. Trials focusing exclusively on perineal tears unrelated to episiotomy, mixed obstetric populations without separate episiotomy data, or complicated episiotomy wounds associated with infection, systemic disease (e.g., diabetes mellitus), or major obstetric complications were excluded when relevant data could not be isolated. Additionally, studies that did not report extractable clinical outcomes related to perineal pain or wound healing, used non-clinical or laboratory settings, or were published in languages other than English were excluded.

Titles and abstracts were screened independently by two reviewers. Full-text articles were assessed independently for eligibility by the same reviewers. Disagreements were resolved through discussion, with consultation of a third reviewer when necessary. No automation tools were used in the study selection process.

### 2.4. Data Extraction

Data extraction was performed independently by two reviewers (A.A.A. and M.S.) using a predefined and piloted data extraction form. Any discrepancies were resolved through discussion, and when necessary, consultation with a third reviewer (A.F.). Extracted data were organized into two main domains: study characteristics and outcomes, and PBM intervention parameters.

For study characteristics and findings, the following items were extracted from each included study: population characteristics (clinical condition), study design, sample size, mean age (±standard deviation), measured outcomes, details of the intervention, main comparator(s), type of control intervention, and a summary of the reported results for pain and/or wound-healing outcomes.

In addition, detailed information on PBM parameters was extracted to allow comparison of dosimetry and treatment protocols across studies. These parameters included the type of light source (e.g., low-level laser therapy, light-emitting diode, or cluster probes), wavelength (nm), output power (mW), spot size (cm^2^), pulse frequency (Hz) when applicable, energy density (J/cm^2^), total energy delivered (J), treatment time per session, session frequency (sessions per week), and total number of treatment sessions. When required data were missing or unclear, corresponding authors were contacted for clarification. If data could not be obtained, this was reported accordingly, and the study was retained for qualitative synthesis when appropriate.

For pain and wound-healing outcomes, post-intervention values were preferentially extracted. When multiple assessment time points were reported, the earliest post-treatment time point was selected to ensure consistency across studies.

### 2.5. Evaluation of Methodological Quality

The methodological quality and risk of bias of the included studies were assessed independently by two reviewers (R.S. and A.A.) using validated, design-specific tools. RCTs were evaluated using the Cochrane Risk of Bias tool version 2 (RoB 2), which assesses bias across five domains: bias arising from the randomization process, bias due to deviations from intended interventions, bias due to missing outcome data, bias in measurement of the outcome, and bias in selection of the reported result. Each domain was judged as low risk, some concerns, or high risk of bias, and an overall risk of bias judgment was assigned according to Cochrane guidance [27,28].

Non-randomized controlled studies were assessed using the Risk of Bias in Non-randomized Studies of Interventions (ROBINS-I) tool. This tool evaluates bias across seven domains, including bias due to confounding, selection of participants, classification of interventions, deviations from intended interventions, missing data, outcome measurement, and selection of the reported result. Overall risk of bias was categorized as low, moderate, or serious risk, based on the highest level of bias identified in any domain.

Disagreements between reviewers were resolved through discussion, and when necessary, consultation with a third reviewer (A.A.). The results of the methodological quality assessment were summarized in tabular form and visually presented using traffic-light plots. Risk of bias judgment was considered when interpreting pooled results and was incorporated into the GRADE assessment of the certainty of evidence.

### 2.6. Quality of Evidence Evaluation

The overall quality of evidence for each outcome was evaluated using the GRADE approach by (M.S.A. and E.K.). The GRADE framework assesses the certainty of evidence across five domains: risk of bias, inconsistency, indirectness, imprecision, and publication bias. Evidence derived from randomized controlled trials (RCTs) was initially rated as high certainty, whereas evidence from non-randomized studies was initially rated as low certainty, and subsequently downgraded or upgraded based on predefined GRADE criteria [29].

Downgrading decisions were based on the presence of serious or very serious limitations in any of the five domains, including methodological limitations identified through RoB 2 or ROBINS-I, substantial statistical heterogeneity, indirectness of populations or interventions, imprecise effect estimates with wide confidence intervals, and suspected publication bias. The certainty of evidence for each outcome was ultimately classified as high, moderate, low, or very low [30]. GRADE assessments were conducted independently by two reviewers, with disagreements resolved by consensus. The results of the GRADE evaluation were summarized in the Summary of Findings tables and used to inform the interpretation of the meta-analysis results and the strength of conclusions.

### 2.7. Meta-Analysis

Studies were eligible for quantitative synthesis if they reported comparable pain or wound-healing outcomes and provided sufficient numerical data to calculate effect sizes. Studies lacking extractable data were included in the qualitative synthesis only. Meta-analyses were performed when at least two studies reported comparable outcomes and sufficient quantitative data were available. Statistical analyses were conducted using Review Manager (RevMan 5.4) software. Continuous outcomes were pooled using mean differences (MD) or standardized mean differences (SMD) with 95% confidence intervals (CI), depending on outcome measurement scales.

For the primary outcome (pain intensity), pooled effect estimates were calculated using SMDs, as included studies employed different pain assessment tools (e.g., Visual Analog Scale (VAS), Numeric Rating Scale (NRS), and Numeric Pain Rating Scale (NPRS)). For the secondary outcome (wound healing), pooled analyses were conducted using Mean Difference, as wound healing was assessed using the same scale (REEDA) score across studies. A random-effects model was applied for all meta-analyses to account for anticipated clinical and methodological heterogeneity among studies.

When outcomes were reported using different pain scales, standardized mean differences were calculated. Scale directions were aligned so that higher values consistently reflected worse pain or poorer wound healing, as appropriate.

For studies with multiple intervention arms (e.g., red and infrared PBM compared with a single control group), each intervention arm was entered separately into the meta-analysis and compared individually with the control group, in accordance with Cochrane recommendations, while avoiding double-counting of participants. When data were reported in formats other than mean and standard deviation, appropriate statistical methods were used to estimate missing parameters where feasible. Studies that did not provide sufficient quantitative data were excluded from the meta-analysis but retained in the qualitative synthesis. Forest plots were generated to visually present pooled effect estimates and individual study effects.

Statistical heterogeneity was assessed using the I^2^ statistic, with I^2^ values of approximately 25%, 50%, and 75% representing low, moderate, and high heterogeneity, respectively. Sensitivity or subgroup analyses were not performed due to the limited number of included studies and substantial heterogeneity. Given the anticipated clinical and methodological heterogeneity, pooled effect estimates derived from random-effects models were interpreted as average effects across studies rather than as precise estimates applicable to all clinical contexts. The results of the meta-analyses were interpreted in conjunction with the risk of bias assessments and GRADE certainty ratings. Subgroup or meta-regression analyses were not conducted due to the limited number of included studies and substantial heterogeneity. Sensitivity analyses were not performed due to the limited number of studies.

### 2.8. Reporting Bias Assessment

Assessment of reporting bias using funnel plots was planned; however, formal evaluation was not feasible due to the small number of studies included in each meta-analysis.

## 3. Results

### 3.1. Study Selection

The electronic search identified 198 records from databases and registers, including PubMed (*n* = 17), Scopus (*n* = 9), PEDro (*n* = 3), Google Scholar (*n* = 150), CINAHL (*n* = 11), and the Cochrane Library (*n* = 8). After the removal of 28 duplicate records, 170 unique records remained for screening.

**Figure 1:**
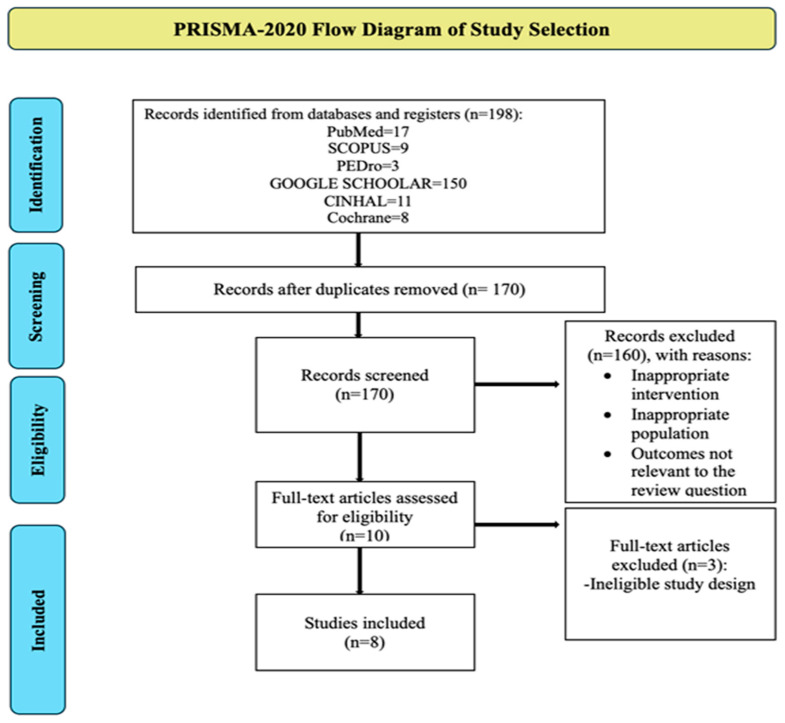
Study flow diagram.

Titles and abstracts of these 170 records were screened, resulting in the exclusion of 160 records due to inappropriate intervention, ineligible population, or outcomes not relevant to the review question. Consequently, 10 full-text articles were retrieved and assessed for eligibility. Of these, three full-text articles were excluded because of ineligible study design, leaving eight studies that met the inclusion criteria and were included in the qualitative synthesis (systematic review). Among the eight included studies, six provided sufficient quantitative data on pain and/or wound-healing outcomes and were therefore included in the meta-analysis. The remaining two studies were excluded from quantitative synthesis due to insufficient or non-extractable data but were retained in the narrative synthesis [22,23,24,25]. A summary of the study selection process is presented in Figure 1 (PRISMA-2020 flow diagram).

Two identified records were excluded after full-text assessment. One article was excluded because it was a systematic review and meta-analysis rather than an original primary study and therefore did not meet the eligibility criteria for inclusion of individual clinical trials [21]. The second study was excluded because it employed a non-comparative design, in which photobiomodulation outcomes were reported only within a single treatment group without comparison to a control or alternative intervention group, precluding estimation of a comparative treatment effect [31].

### 3.2. Data Extraction

Across all included studies, the population was largely homogeneous, consisting of postpartum women who underwent mediolateral episiotomy [11,22,23,24,25,32,33,34]. Most trials explicitly restricted inclusion to women with right mediolateral episiotomy, ensuring anatomical and clinical consistency [23,24,33]. A single study broadened eligibility to include women with either episiotomy or second-degree perineal laceration, introducing minor clinical heterogeneity related to wound type and tissue trauma severity [34]. No study included instrumental deliveries, third- or fourth-degree tears, or cesarean sections, supporting reasonable comparability across trials (Table 1).

RCTs constituted the majority of included studies, including double-blind, triple-blind, and parallel-group RCTs [11,23,24,32,33,34]. Two non-randomized controlled studies employed comparative studies [22,25], which increases susceptibility to selection bias and limits internal validity. Blinding was inconsistently applied; while several trials implemented double- or triple-blinding, earlier studies often lacked clear reporting of allocation concealment or assessor blinding (Table 1).

Sample sizes ranged substantially, from small pilot studies [22,25] to adequately powered RCTs enrolling over 100 participants [23]. Early or exploratory studies [22,25] included fewer than 20 participants, limiting statistical power. Larger trials such as Santos (2012a, *n* = 114) and Gondim (2025, *n* = 60) provided more robust estimates of treatment effects [23,34]. Participant age was remarkably consistent across studies, with mean ages typically ranging from early to mid-20s, reflecting a relatively young postpartum population. Where reported, standard deviations were narrow, indicating low age dispersion. Several studies did not report age, representing incomplete demographic reporting but are unlikely to substantially affect outcome comparability [22,25].

Pain was assessed consistently across studies using validated pain scales, including the Numeric Pain Rating Scale (NPRS) [11,23,24,33,34], the Visual Analog Scale (VAS) [32], and the McGill Pain Questionnaire and Short-Form McGill Pain Questionnaire (SF-MPQ) [34]. This consistency supports quantitative synthesis. Only two studies used an ordinal level for pain evaluation (mild, moderate, severe) [22,25]. Pain assessment focused on early postpartum periods, aligning with clinical relevance.

Wound healing was commonly evaluated using the (REEDA) scale, assessing redness, edema, ecchymosis, discharge, and approximation [11,24,32,33,34]. An earlier study additionally reported clinical signs such as tenderness and exudate without standardized scales [22]. Overall, wound-healing assessment methods were moderately consistent, with REEDA being the dominant tool.

All included studies employed low-level laser therapy (LLLT) as the PBM modality. PBM was predominantly diode-based systems, which are standard in clinical PBM applications for soft tissue healing and pain modulation [22,23,24,25,32,33]. This consistency indicates that the available evidence relates exclusively to low-intensity PBM, limiting extrapolation to other light-based modalities. Red and infrared wavelength bands were used across studies. Red light centered around 660 nm, and infrared (IR) light ranged from 780 to 808 nm. Several studies used single wavelengths (either red or IR), while more recent trials [11,34] applied combined red + infrared protocols (660 nm + 808 nm) within the same treatment session.

There was substantial variability in output power across studies. Studies used low-power outputs: 15–35 mW [23,24,32], moderate outputs: 100 mW [11,34], and high reported outputs: 0.6–1.0 W [22,25], although still delivered under LLLT classifications due to short exposure times and limited spot sizes. All studies applied PBM in a continuous wave mode; no pulsed protocols or frequency-modulated emissions were used [11,22,23,24,25,32,33,34]. This represents a consistent application approach across trials.

Spot sizes were small and localized, particularly in RCTs that used point-by-point irradiation over the episiotomy wound margins. Reported spot sizes ranged from 0.04 cm^2^ to approximately 0.5 cm^2^, although several studies incompletely reported this parameter [22,25]. Most protocols applied PBM to multiple points along the wound line, typically three [23,24] to nine points [33], reflecting attempts to cover the entire incision length. Energy density demonstrated the widest range of variations across studies. Studies used lower fluences (3–9 J/cm^2^) [11,23,24], moderate fluences (20 J/cm^2^) [25], or very high fluences: 50–100 J/cm^2^ [34]. Total energy delivered per session ranged from 0.45 J in early low-dose studies to 16 J per session in more recent trials. Several studies did not report total energy explicitly, limiting direct dose comparisons [11,22,32].

Across the randomized controlled trials, PBM protocols were generally characterized by low energy densities and limited treatment exposure. Most RCTs applied energy densities ≤ 9 J/cm^2^ per point and delivered no more than one to three treatment sessions, often within a single day or over a short postoperative period. Higher fluences and repeated-session protocols were uncommon in randomized designs and were more frequently observed in small non-randomized studies. This pattern highlights a predominance of low-dose, short-duration PBM protocols among trials contributing to the quantitative synthesis.

The treatment time per point ranged from 10 to 60 s, depending on power output and target fluence. Single-session protocols were used in several RCTs [11,23,34], primarily targeting immediate postpartum pain. Multiple-session protocols (2–4 sessions) were applied in earlier or comparative studies [22,24,25,32], often over several days. The total number of sessions ranged from 1 to 4 sessions, with no study exceeding one week of treatment.

Comparator interventions varied and included sham laser [23,24,33,34], standard care (antibiotics ± NSAIDs) [22,25], and cryotherapy [11,32]. The frequent use of sham controls strengthens internal validity, while active comparators provide clinically meaningful comparisons (Table 2).

Pain reduction was observed across almost all groups, including controls, reflecting natural postpartum recovery. However, differences between groups were inconsistent. Several trials reported no significant superiority of PBM over sham [23,24,33,34]. In contrast, Cristine (2024) demonstrated greater pain reduction with PBM compared to cryotherapy [11]. Limited studies generally favored PBM, though with higher risk of bias [22]. Improvements in wound healing were generally reported in both intervention and control groups, with limited evidence of PBM superiority. Most RCTs found no significant between-group differences in REEDA scores, whereas smaller comparative studies suggested faster healing with PBM [11,24,32,33,34].

Overall, the included studies demonstrate consistent population characteristics and reasonable methodological diversity, with pain as the primary outcome and wound healing as a secondary outcome. While PBM appears safe and well-tolerated, evidence for its superiority over sham or standard care remains inconsistent, particularly in well-designed RCTs. These findings inform both the narrative synthesis and forthcoming meta-analysis.

### 3.3. Risk of Bias Assessment

The risk of bias for the RCTs was evaluated using the Cochrane Risk of Bias tool version 2 (RoB 2) across five domains: randomization process, deviations from intended interventions, missing outcome data, outcome measurement, and selection of the reported result. Overall, one trial was judged to be at low risk of bias [24], two trials were judged as having some concerns [33,34], and three trials were judged to be at high risk of bias [11,23,32]. High-risk judgments were predominantly driven by limitations related to deviations from intended interventions and outcome measurement, particularly in studies using active comparators and lacking adequate blinding.

Most RCTs demonstrated a low risk of bias arising from the randomization process, supported by appropriate sequence generation and baseline comparability between intervention groups [11,23,24,33]. In contrast, Ashwini and Mahishale (2015) showed some concerns related to randomization, reflecting limited reporting of allocation concealment procedures [32]. The Constant et al. (2024) study was judged at high risk of bias in this domain due to insufficient methodological detail regarding random sequence generation and allocation concealment, raising concerns about potential selection bias [11].

**Figure 2:**
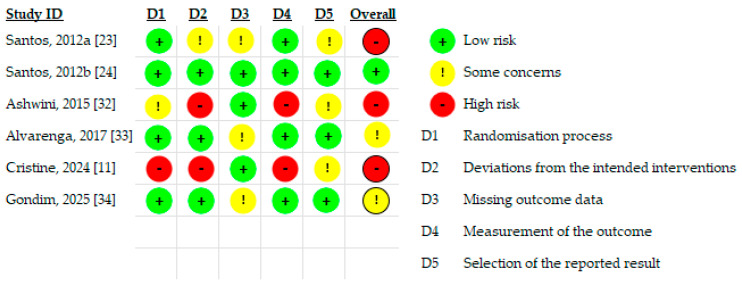
Risk of bias assessment for RCT (RoB2) (Studies [11,23,24,32,33,34]).

Bias due to deviations from intended interventions varied across studies. Three studies were judged at low risk, supported by adequate blinding procedures and adherence to prespecified intervention protocols [24,33,34]. In contrast, one trial raised some concerns, likely related to the single-dose PBM design and the potential for behavioral responses within the trial context, despite participant blinding [23]. Two trials were rated at high risk of bias in this domain, primarily due to the use of active comparators (ultrasound and cryotherapy, respectively), absence of blinding of participants and personnel, and an increased likelihood of performance bias influencing subjective pain outcomes [11,32] (Table 3 and Figure 2).

Across the included RCTs, missing outcome data were generally minimal and did not differ meaningfully between groups. Most studies were judged at low risk of bias in this domain. However, some concerns were noted in three studies due to incomplete reporting of attrition handling or the absence of explicit intention-to-treat analyses [23,33,34]. Nevertheless, attrition rates were low and unlikely to have materially influenced the study results.

Outcome measurement was judged to be at low risk of bias in the majority of studies, reflecting the use of validated pain assessment tools (e.g., NPRS, VAS, McGill Questionnaires) and standardized wound-healing assessments such as the REEDA scale. In contrast, two studies were rated at high risk of bias in this domain, mainly due to lack of assessor blinding and reliance on subjective pain outcomes when comparing PBM with active physical modalities, increasing the likelihood of detection bias [11,32].

Most studies demonstrated low risk or some concerns regarding selective reporting. None of the trials had a publicly available protocol or trial registration, resulting in some concerns about selective reporting in several studies [11,23,32]. However, the outcomes reported were generally consistent with those described in the methods sections, and no clear evidence of selective outcome reporting was identified (Figure 2).

**Figure 3:**
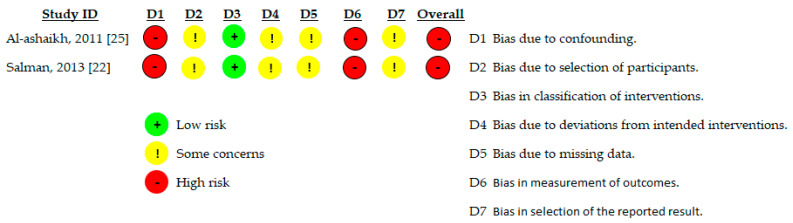
Risk of bias in non-randomized studies of interventions (ROBINS-I) (Studies [22,25]).

Two non-randomized controlled studies were assessed using the ROBINS-I tool and were judged to be at serious risk of bias overall [22,25]. Major sources of bias included confounding due to non-random allocation and co-interventions, lack of blinding, and subjective outcome measurement. Consequently, findings from these studies were interpreted with caution and considered supportive rather than confirmatory (Table 4 and Figure 3).

As illustrated in Figure 2 and Figure 3, the most frequent sources of bias across the included studies related to deviations from intended interventions and outcome measurement. In several trials, lack of adequate blinding, particularly when active comparators were used, increased the risk of performance and detection bias for subjective pain outcomes. In addition, incomplete reporting of allocation concealment and absence of pre-registered protocols contributed to concerns regarding selective reporting. These recurring limitations across key domains explain the predominance of high or serious risk of bias judgments and underpin the downgrading of the certainty of evidence to very low.

### 3.4. Quality of Evidence Assessment

The certainty of evidence for pain outcomes was rated as very low. Although data were pooled from six studies including 434 participants, the evidence was downgraded due to serious study limitations, serious inconsistency, and serious imprecision. Methodological concerns primarily related to inadequate reporting of allocation concealment, lack of blinding of participants, therapists, or outcome assessors, and inclusion of studies with high or serious risk of bias. Inconsistency was evident due to substantial statistical heterogeneity (I^2^ > 75%) across studies. Imprecision was present as the pooled effect estimate was based on relatively small sample sizes and wide confidence intervals, which crossed the minimally important difference. Potential publication bias was considered possible because funnel plots were underpowered (<10 studies), although no formal downgrading was applied. The pooled effect estimate showed a small effect size (SMD = 0.04; 95% CI −0.60 to 0.68), with no clear direction of effect favoring PBM, indicating uncertainty regarding its analgesic benefit (Table 5).

Similarly, the certainty of evidence for wound-healing outcomes was rated as very low. Evidence was derived from six studies comprising 282 participants and was downgraded due to serious study limitations, serious inconsistency, and serious imprecision. High risk of bias across several included studies, variability in wound-healing assessment methods, and substantial heterogeneity contributed to the inconsistency rating. Imprecision was driven by limited sample size and wide confidence intervals around the pooled effect estimate. Publication bias was again considered possible due to the small number of contributing studies. The pooled analysis demonstrated a large effect size (MD = 0.94; 95% CI −0.69 to 2.56); however, the confidence interval crossed the line of no effect, resulting in no clear direction of effect favoring PBM.

### 3.5. Meta-Analysis

A meta-analysis was conducted to evaluate the effect of PBM on pain intensity following episiotomy. Data from six studies [11,23,24,32,33,34] involving 434 participants (PBM: *n* = 215; control: *n* = 219) were pooled using a random-effects model due to substantial clinical and methodological heterogeneity. One included RCT evaluated two active PBM (red and infrared wavelengths) compared with a single control group (sham laser) for perineal pain intensity measured using the NPRS. Each PBM intervention arm was compared separately with the control group, and the resulting effect estimates were entered individually into the meta-analysis.

**Figure 4:**
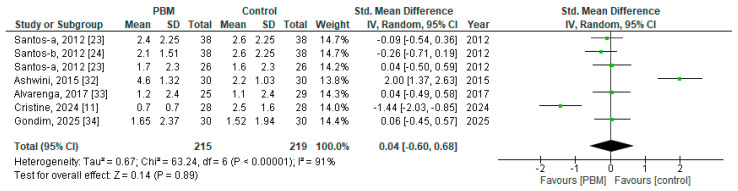
Forst plot for pain outcomes (Studies [11,23,24,32,33,34]).

The pooled analysis demonstrated no significant difference in pain intensity between the PBM and control groups (SMD = 0.04; 95% CI −0.60 to 0.68; *p* = 0.89). The effect size was trivial, and the confidence interval crossed the line of no effect, indicating no clear analgesic benefit of PBM compared with control interventions. Statistical heterogeneity was considerable (I^2^ = 91%; τ^2^ = 0.67; χ^2^ = 63.24, *p* < 0.00001), suggesting substantial variability in effect estimates across studies. Individual trials showed inconsistent directions of effect, with some studies favoring PBM, others favoring control, and several showing minimal or null effects. This variability likely reflects differences in PBM parameters, comparator interventions, treatment dosage, and methodological quality. Overall, the pooled findings indicate that PBM therapy did not provide a statistically or clinically meaningful reduction in pain intensity following episiotomy when compared with control conditions (Figure 4). There is currently no established minimally important difference (MID) for standardized pain outcomes specifically in the context of post-episiotomy PBM interventions. Consequently, clinical interpretation of the pooled effect relies on the magnitude and precision of the estimated effect and its confidence interval rather than comparison with a predefined MID.

**Figure 5:**
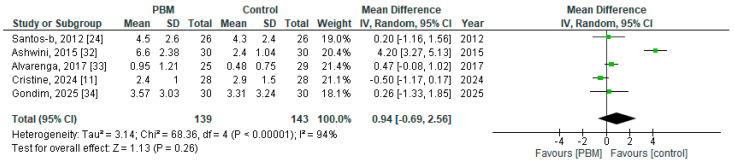
Forst plot for wound-healing outcomes (Studies [11,24,31,32,33]).

A meta-analysis was performed to evaluate the effect of PBM therapy on wound healing following episiotomy, measured using the REEDA scale. Data from five studies [11,24,32,33,34] comprising 282 participants (PBM: *n* = 139; control: *n* = 143) were pooled using a random-effects model, given the marked clinical and methodological heterogeneity among trials. The pooled analysis showed no statistically significant difference in wound-healing outcomes between the PBM and control groups (MD = 0.94; 95% CI −0.69 to 2.56; *p* = 0.26). Even though the point estimate favored PBM, the confidence interval crossed the line of no effect, indicating no clear evidence of superior wound healing associated with PBM compared with control interventions (Figure 5). Similarly, no validated MID has been established for changes in REEDA scores following episiotomy, which limits the ability to determine whether the observed confidence interval reflects a clinically meaningful difference.

Heterogeneity among studies was considerable (I^2^ = 94%; χ^2^ = 68.36, *p* < 0.00001), reflecting substantial variability in treatment effects across trials. Individual studies demonstrated inconsistent findings, with some reporting improvements in REEDA scores following PBM and others showing minimal or no between-group differences. This heterogeneity may be attributable to differences in PBM parameters (wavelength, energy density, number of sessions), comparator treatments, timing of outcome assessment, and overall methodological quality. Due to the limited number of included studies, no analyses exploring sources of heterogeneity were conducted and no sensitivity analyses were performed. Overall, the meta-analysis indicates that PBM therapy did not result in a statistically or clinically significant improvement in wound healing after episiotomy when compared with control conditions.

### 3.6. Reporting Bias

Formal assessment of reporting bias was not performed because fewer than ten studies were included in each meta-analysis.

## 4. Discussion

This systematic review and meta-analysis synthesized evidence from eight studies evaluating the effects of PBM therapy on pain and wound healing after episiotomy, with six studies contributing to quantitative synthesis. Despite a clear biological rationale for PBM, pooled analyses demonstrated no statistically significant benefit for either pain reduction or wound healing as compared to control interventions. These findings were accompanied by considerable heterogeneity (I^2^ > 90%) and a very low certainty of evidence, driven primarily by methodological limitations, inconsistency, and imprecision. Importantly, both randomized and non-randomized studies contributed to the evidence base, with the majority of trials judged to be at high or serious risk of bias, which substantially limits confidence in the pooled estimates.

Although one included study enrolled participants with either episiotomy or second-degree perineal tears, this was considered unlikely to materially bias the findings. Both conditions involve injury to the perineal skin and underlying musculature, are managed similarly in routine obstetric practice, and typically exhibit comparable early healing patterns. Consequently, inclusion of this study was judged to introduce only minor clinical heterogeneity and did not warrant exclusion or separate qualitative analysis.

A major factor underlying the lack of statistically significant findings in the present review is the overall poor methodological quality of the included studies. Only one randomized trial was judged to be at low risk of bias, while several trials raised some concerns or were classified as high risk under RoB 2. Additionally, the two non-randomized controlled studies were judged to be at serious risk of bias using ROBINS-I, mainly due to confounding, lack of allocation concealment, and inadequate blinding. Similar concerns have been highlighted in earlier reviews of PBM for wound healing, which consistently report small sample sizes, insufficient blinding, and suboptimal reporting of laser parameters as major limitations of the literature [22,25]. These methodological weaknesses likely diluted true treatment effects and contributed to imprecise pooled estimates.

The substantial heterogeneity observed in both pain and wound-healing meta-analyses represents another critical explanation for the absence of significant pooled effects. Prior systematic reviews consistently emphasize that PBM outcomes are highly sensitive to treatment parameters, including wavelength, energy density, power output, timing of application, and number of sessions. In the included episiotomy trials, there was wide variation in PBM protocols, with energy densities ranging from subtherapeutic to potentially supra-therapeutic levels, inconsistent session frequency, and differences in timing relative to delivery. This heterogeneity is consistent with earlier narrative and meta-analytic reviews, which identified lack of protocol standardization as a central reason for inconsistent clinical outcomes across PBM trials, even when biological mechanisms are well established. In the presence of very high statistical heterogeneity, narrative synthesis of individual study findings may be more informative than reliance on pooled point estimates alone, particularly for understanding the context-specific effects of PBM protocols.

A critical concept repeatedly highlighted in the broader PBM literature is the biphasic dose–response relationship, whereby low-to-moderate energy densities stimulate tissue repair, while higher doses may result in diminished or null effects [20]. Several high-quality reviews have reported that optimal wound-healing responses are most consistently observed with energy densities between approximately 1 and 10 J/cm^2^, whereas higher doses may negate beneficial effects [21,35,36]. In the present review, many included studies either failed to report complete dosimetric details or employed heterogeneous dosing protocols, making it difficult to ascertain whether PBM was delivered within the optimal therapeutic window. This likely contributed to the neutral pooled effects observed in both pain and wound-healing outcomes.

The experimental and clinical PBM literature consistently demonstrates a biphasic dose–response relationship, whereby insufficient energy densities fail to elicit biological effects, while excessive doses may inhibit cellular activity and delay tissue repair. In the included episiotomy trials, several studies employed very low energy densities (≤3–4 J/cm^2^) or single-session protocols that may have been subtherapeutic, whereas others applied relatively high fluences (≥50–100 J/cm^2^) that could exceed the optimal stimulatory range. This wide dispersion of dosimetric parameters suggests that some studies with neutral or negative results may not have delivered PBM within an effective therapeutic window. Consequently, the absence of significant pooled effects in this meta-analysis may reflect suboptimal dosing rather than true ineffectiveness of PBM. Future trials should explicitly target and justify PBM dosimetry within established therapeutic ranges and report parameters comprehensively to allow replication and meaningful synthesis.

The lack of statistically significant findings observed in the present review may, in part, be attributed to limitations in treatment protocols and suboptimal prescription of laser dose and treatment frequency in the included studies. All included trials employed LLLT using a point-based application, with a limited number of irradiation points and relatively short exposure times and energy densities per point [23,24,25]. Such parameters may have been insufficient to elicit a clinically meaningful PBM response in acute perineal wounds.

Most randomized controlled trials included in this review employed point-by-point irradiation along the episiotomy incision margins, rather than scanning or wide-area application techniques. While point-based delivery allows precise dosing at selected sites, it may result in incomplete coverage of the entire wound area, particularly for longer or irregular incisions. In contrast, scanning or wide-area application techniques have been proposed to provide more uniform energy distribution across surgical wounds; however, direct comparisons between point-by-point and scanning techniques are lacking in the episiotomy literature. Consequently, it remains unclear whether point-based application is superior to, equivalent to, or less effective than wide-area approaches. The reliance on point-by-point application in most trials may therefore have limited overall energy delivery and contributed to the inconsistent clinical effects observed. Future studies should explicitly compare application techniques and ensure adequate wound coverage when evaluating PBM efficacy after episiotomy.

An important clinical consideration emerging from this synthesis is the nature of episiotomy as an acute, clean surgical wound occurring in otherwise healthy tissue with a strong inherent capacity for rapid healing. In this context, the expected incremental benefit of PBM over natural physiological recovery is likely small, particularly when compared with chronic or compromised wounds such as diabetic foot ulcers, venous ulcers, or pressure injuries. When this limited potential for additive benefit is combined with suboptimal or heterogeneous PBM protocols and methodological limitations of the existing trials, null or inconclusive findings become more readily explainable. This distinction between acute surgical wounds and chronic non-healing wounds is clinically important and cautions against extrapolating PBM efficacy across fundamentally different wound types.

The choice of comparator intervention represents an additional factor that may have influenced the detection of PBM-specific effects. Several included trials compared PBM with active “conventional care” interventions, such as cryotherapy [11], ultrasound [31], systemic analgesics, anti-inflammatory medications, or standard postpartum management [22,25]. These interventions are themselves known to reduce pain and inflammation and to support natural wound healing. Consequently, the use of active comparators may have attenuated between-group differences and reduced the likelihood of detecting an additional or incremental benefit attributable solely to PBM. This is particularly relevant in the context of acute episiotomy wounds, where spontaneous recovery is rapid and baseline pain levels decline naturally over time. While active comparators enhance clinical relevance, they may obscure modest PBM effects when compared with sham or no-treatment controls [23,24,32,33]. This methodological consideration should be taken into account when interpreting neutral findings and designing future trials, which may benefit from including both sham and active comparator arms to better isolate the specific contribution of PBM.

Although the current review included relatively recent trials, the type of PBM devices used remained largely outdated, with limited technological advancement in beam delivery, power output, or treatment coverage [34]. Notably, none of the included studies investigated high-intensity laser therapy (HILT), which represents a more advanced form of PBM and has been increasingly adopted in contemporary wound management. Emerging evidence indicates that HILT produces deeper tissue penetration, greater energy delivery, and enhanced biological effects, and has demonstrated promising outcomes in accelerating wound healing in conditions such as diabetic foot ulcers, neuropathic ulcers, and improving delayed healing following cesarean section in diabetic women [37,38,39,40].

Evidence from previous meta-analyses demonstrates that PBM is more consistently effective in chronic or slow-healing wounds, such as diabetic foot ulcers, venous ulcers, and pressure injuries, than in acute surgical wounds [38,41,42]. Episiotomy represents a clean, acute surgical wound in generally healthy tissue, with rapid physiological healing already expected. The relative benefit of PBM may be smaller and harder to detect, particularly when baseline pain levels are low and follow-up periods are short. This context-dependent effect has been explicitly noted in recent meta-analyses that included episiotomy as a subgroup and similarly reported non-significant pain outcomes despite favorable results in other wound types [21].

The absence of a significant analgesic effect of PBM in the present meta-analysis may also relate to the timing of outcome assessment. Experimental and clinical evidence suggests that PBM exerts its strongest anti-inflammatory and analgesic effects when applied during the early postoperative inflammatory phase, when nociceptive mediators and local edema are most pronounced. In the included trials, there was notable variability in the time elapsed between episiotomy repair and the first PBM session, with some studies applying PBM within the first hours postpartum, while others initiated treatment one or more days later. Delayed application may coincide with the natural resolution of acute inflammation and pain, potentially diminishing the observable treatment effect and contributing to neutral findings. This variability in treatment timing represents an additional source of clinical heterogeneity and may partly explain the inconsistent analgesic effects observed across studies. Future trials should standardize and clearly report the timing of PBM initiation relative to suturing to better delineate its role in acute pain modulation.

Evidence from surgical and periodontal meta-analyses suggests that PBM exerts its strongest analgesic effects during the early postoperative inflammatory phase, with diminishing effects over time [35]. In several episiotomy trials, pain was assessed at later postpartum time points, when pain levels naturally decline, potentially masking any early transient benefits of PBM [24,25,32]. Additionally, variability in pain measurement tools (VAS vs. NRS) and differences in co-interventions (e.g., analgesic use) may have further contributed to inconsistent findings.

Variability in skin pigmentation represents a further theoretical factor that could influence PBM effectiveness, as melanin is known to absorb visible and near-infrared light and may alter photon penetration depth. Differences in skin color could therefore modulate the amount of energy reaching deeper tissues and potentially affect clinical response. However, none of the included studies reported participants’ skin color or optical tissue characteristics, precluding any stratified analysis or evaluation of this factor in the present review. Moreover, the majority of included trials were conducted in relatively homogeneous populations within single geographic regions, further limiting the variability that could be examined. While differences in skin pigmentation cannot be excluded as a contributing source of heterogeneity, the available evidence does not allow this hypothesis to be tested.

The very low certainty of evidence for both pain and wound healing indicates that the true effect of PBM after episiotomy may differ substantially from the pooled estimates. While extensive preclinical and clinical evidence supports the biological plausibility of PBM in promoting tissue repair and modulating inflammation, the current episiotomy-specific evidence base remains methodologically weak and clinically heterogeneous.

Although this review included both randomized and non-randomized controlled studies to provide a comprehensive overview of the available literature, the interpretation of findings should primarily be guided by evidence from RCTs. Notably, the neutral effects observed in the meta-analyses were largely driven by RCTs with sham or active comparators, whereas studies reporting the favorable effects of PBM were predominantly non-randomized and judged to be at serious risk of bias. Consequently, conclusions regarding the clinical effectiveness of PBM after episiotomy should rely mainly on RCT evidence, which currently does not demonstrate a clear benefit over control interventions.

Future trials evaluating PBM after episiotomy should prioritize rigorous randomized designs, adequate sample sizes, standardized PBM protocols within established therapeutic dose ranges, and early postoperative outcome assessment. Detailed reporting of laser parameters is essential to allow reproducibility and meaningful synthesis. Given the promising findings in other surgical and chronic wound contexts, well-designed episiotomy-specific trials remain justified, but current evidence does not support definitive clinical recommendations. In addition, future trials should be prospectively registered with detailed, pre-specified PBM parameters to enhance transparency and reproducibility. Adoption of standardized core outcome sets, such as validated pain scales assessed at fixed early postpartum time points and repeated REEDA assessments across multiple postpartum days, would further reduce heterogeneity and facilitate meaningful comparison and synthesis across studies.

Based on mechanistic evidence and the prior PBM literature, an optimal trial protocol would include early initiation of PBM within the first 6–12 h following episiotomy suturing, targeting the acute inflammatory phase. A red or combined red–infrared wavelength range (e.g., 660 nm ± 808 nm) may be appropriate to balance superficial tissue coverage and deeper penetration. Energy density should be delivered within established therapeutic windows (approximately 4–10 J/cm^2^ per point), avoiding both subtherapeutic and potentially inhibitory doses. Treatment should ensure adequate wound coverage using either closely spaced point-by-point irradiation or controlled scanning techniques, with total energy adjusted to incision length. Repeated sessions (e.g., 2–3 sessions over the first 48–72 h postpartum) may be preferable to single-dose protocols. Trials should employ sham-controlled designs alongside standardized conventional care, with early pain outcomes assessed within 24–48 h. Comprehensive reporting of dosimetry, application technique, timing, and patient characteristics is essential to enable replication and definitive synthesis.

## 5. Conclusions

This systematic review and meta-analysis synthesized the available evidence on the effectiveness of PBM therapy for the management of perineal pain and wound healing following episiotomy. Based on eight included studies, with six contributing to quantitative synthesis, the pooled analyses demonstrated no statistically significant benefit of PBM compared with control interventions for either pain reduction or wound-healing outcomes. These findings were accompanied by substantial heterogeneity, a predominance of studies with high or serious risk of bias, and a very low certainty of evidence, indicating that the true effect of PBM may be substantially different from the estimated effects.

The absence of significant pooled effects appears to be driven by methodological limitations, clinical and dosimetric heterogeneity, and suboptimal PBM protocols, including limited treatment coverage, insufficient energy delivery, and reliance on older low-level laser devices. While the extensive experimental and clinical literature supports the biological plausibility of PBM in tissue repair and pain modulation, the current episiotomy-specific evidence base remains insufficient to support definitive clinical recommendations. Importantly, these findings do not exclude the possibility of a small beneficial effect of PBM; however, any such effect remains uncertain and is likely dependent on treatment parameters, timing of application, and study design, none of which are yet sufficiently standardized.

Future research should prioritize well-designed, adequately powered RCTs with standardized and clearly reported PBM parameters, early postoperative outcome assessment, and exploration of advanced PBM approaches, such as high-intensity laser therapy. Until such evidence is available, the routine use of PBM therapy for post-episiotomy pain and wound management should be considered experimental and applied with caution.

This systematic review and meta-analysis has several methodological strengths. First, the review was prospectively registered in PROSPERO, enhancing transparency and reducing the risk of selective reporting. Second, the review followed PRISMA-2020 guidelines and applied rigorous, design-specific risk of bias tools (RoB 2 and ROBINS-I), allowing for a nuanced appraisal of methodological quality across randomized and non-randomized studies. Third, the use of random-effects meta-analysis accounted for expected clinical and methodological heterogeneity, and outcomes were analyzed using appropriate effect measures (SMD for pain and MD for wound healing). Finally, the review provides a comprehensive synthesis of PBM parameters, offering valuable insight into protocol variability and potential sources of inconsistent effects across studies.

Several limitations should be acknowledged. The overall certainty of evidence was very low, largely due to high or serious risk of bias in most included studies, substantial heterogeneity, and imprecision of pooled estimates. The small number of eligible studies limited the ability to perform subgroup or sensitivity analyses, such as comparisons by wavelength, dose, or number of treatment sessions. In addition, there was considerable clinical and dosimetric heterogeneity in PBM protocols, including variation in wavelength, energy density, treatment duration, and timing of outcome assessment, which may have obscured potential treatment effects. The inclusion of non-randomized controlled studies further reduced confidence in the pooled results. Even though gray literature sources were searched to minimize publication bias, this strategy may have increased the likelihood of including studies that had not undergone formal peer review, potentially affecting the overall reliability of the evidence. Finally, restriction to English-language publications may have introduced language bias, and the small number of included studies precluded formal assessment of publication bias.

## Figures and Tables

**Table 1:** Summary of included studies’ characteristics and findings.

Authors	Population (Disease)	Study Design	Sample Size	Mean Age ± SD	Measured Outcomes	Groups	Comparator	Result Summary
**Al-ashaikh, 2011 [25]**	Postpartum women with mediolateral episiotomy	Three-group comparative study	19	NR	Pain, tenderness, redness, edema, exudate, wound healing time.	G1 (Control): 7 antibiotics Group 2 (Laser + antibiotics): 6 Group 3 (Laser only): 6	Systemic antibiotics only, no laser.	Laser groups showed faster wound healing (7 days vs. 9–11 days in control). Pain and tenderness improved after first session in both laser groups. Control group showed slow healing; one case developed dehiscence.
**Santos, 2012a [23]**	Right mediolateral episiotomy	Double-blind, three-arm RCT.	114 38 each	Control: 22.5 ± 4.1 Red: 23.3 ± 4.5 Infrared: 22.6 ± 4.7	Perineal pain intensity: NPRS. Patient satisfaction	G1: Control (sham) G2: Red laser (660 nm) G3: Infrared laser (780 nm)	Sham laser	All groups showed significant perineal pain reductions with no significant differences between groups. LLLT did not outperform sham treatment. Single-dose PBM protocol was insufficient to produce analgesic effect.
**Santos, 2012b [24]**	Mediolateral episiotomy	Double-blind RCT (parallel groups).	52 26 each	Control: 23.9 ± 5.0 Experimental: 22.8 ± 4.7	Pain: NPRS. Wound healing: REEDA scale	G1: Control (sham) G2: Red laser (660 nm)	Sham laser	Significant pain reductions in the experimental group with no significant differences between groups. REEDA scores showed no significant group differences. LLLT (3 × 660 nm low dose) did not improve pain or healing.
**Salman, 2013 [22]**	Mediolateral episiotomy	Comparative study	*n* = 18 Control: *n* = 8 Laser: *n* = 10	NR	Pain (mild–severe) Tenderness Redness Edema Discharge	G1: Control G2: Diode laser (LLLT) 790–805 nm	Systemic antibiotics + NSAIDs	Laser group showed lower proportions of pain, tenderness, redness, edema, and discharge at all follow-up time points. diode laser enhances healing and reduces symptoms.
**Ashwini, 2015 [32]**	Mediolateral episiotomy	RCT (2-arm parallel group)	60 30 each	G1: 23.4 ± 3.92 G2: 24.0 ± 2.06	Pain: VAS Wound healing: REEDA score.	G1: Cryogel Pad + US. G2: Cryogel Pad + LLLT	Cryogel Pad (10 min.) + US (3 MHz, 0.5 W/cm^2^).	Pain decreased significantly within groups. Ultrasound group improved more than LLLT in pain and wound healing.
**Alvarenga, 2017 [33]**	Right mediolateral episiotomy	Triple-blind RCT, parallel groups.	54 G1: 25 G2: 29	G1: 22.6 ± 5.0 G2: 22.0 ± 3.5	Pain: NPRS Wound: REEDA	G1: LLLT (808 nm) G2: Sham	Sham laser	Pain was higher in LLLT group than placebo with no difference between groups in pain or REEDA. LLLT did not reduce pain or improve healing after episiotomy.
**Cristine, 2024 [11]**	Episiotomy	RCT	56 28 each	24.6 ± 5.6 years	Pain: NPRS Wound: REEDA Pain quality: McGill Pain Questionnaire	G1: LLLT (660 nm+ 808 nm) G2: Cryotherapy	Cryotherapy latex glove filled with crushed ice	PBM produced significantly greater pain reduction than cryotherapy. REEDA score improved in both groups with no significant between-group difference. No adverse effects reported.
**Gondim, 2025 [34]**	Episiotomy OR second-degree perineal laceration	Double-blind, RCT two-center	60 30 in each	25.6 ± 5.1 years	Pain: NRS, VAS, Short-Form McGill Pain Questionnaire Wound: REEDA scale.	G1: PBM G2: Sham	Sham laser	No significant between-group differences. Both groups improved over time consistently with natural healing

**Abbreviations:** PBM: photobiomodulation, LLLT: low-level laser therapy, RCT: randomized controlled trial, NR: not reported, VAS: visual analog scale, NRS/NPRS: numeric rating scale/numeric pain rating scale, REEDA: redness, edema, ecchymosis, discharge, approximation scale, US: ultrasound, NSAIDs: non-steroidal anti-inflammatory drugs.

**Table 2:** Summary of photobiomodulation parameters in included studies.

	Laser Type (LLLT/LED/Cluster)	Wavelength (nm)	Power (mW)	Spot Size (cm^2^)	Pulse Frequency (Hz)	Energy Density (J/cm^2^)	Total Energy (Joule)	Treatment Time Session	Frequency (Sessions per Week)	Total Sessions
**Al-ashaikh, 2011 [25]**	LLLT, diode laser (GaAlAs)	790–805 nm	1 W	0.8 cm	Continuous mode	19.9 J/cm^2^ per spot	NR	10 s per spot	Every other day	4 sessions
**Santos, 2012a [23]**	LLLT (diode laser)	660–780 nm	35 mW	0.04 cm^2^	Continuous mode	8.8 J/cm^2^	1.05 J 0.35 J/point for 3 points	10 s per point for 3 points	1 session	1 session
**Santos, 2012b [24]**	LLLT (diode laser)	660 nm	15 mW	0.04 cm^2^	Continuous mode	3.8 J/cm^2^ 0.15 J/point	0.45 J 0.15 J/point for 3 points	10 s per point for 3 points	1 daily session	3 sessions
**Salman, 2013 [22]**	Diode laser (LLLT)	790–805 nm	0.6 W 1.19 W/cm^2^	8 mm	Continuous mode	NR	NR	NR	2 sessions every 4 days	2 sessions
**Ashwini, 2015 [32]**	Diode laser (LLLT)	660 nm	15 mW	3.8 J/cm^2^	Continuous mode	NR	NR	30 s	1 per day × 3 days	3 sessions
**Alvarenga, 2017 [33]**	Diode LLLT (Class 3B)	808 nm	20 mW	5 J/cm^2^	Continuous	NR	9 points	NR	1 per day × 3 days	3 sessions
**Cristine, 2024 [11]**	LLLT	(660 nm + 808 nm)	100 mW	3 J/cm^2^ + 6J/cm^2^	Continuous	NR	NR	30 +60 s	1 session	1 session only
**Gondim, 2025 [34]**	Red + IR	(660 nm + 808 nm)	100 mW	0.04 cm^2^	Continuous	50 J/cm^2^ for red and 100 J/cm^2^ for IR	16 J per session	20–40 sec/point	1 session	1 session only

**Abbreviations:** LLLT, low-level laser therapy; LED, light-emitting diode; IR, infrared; nm, nanometer; mW, milliwatt; W, watt; cm^2^, square centimeter; Hz, hertz; J, joule; J/cm^2^, joules per square centimeter; NR, not reported.

**Table 3:** Risk of bias assessment for RCT evaluated by (RoB2).

Study	Randomization	Deviations from Intended Interventions	Missing Data	Outcome Measurement	Reporting Selection	Overall Judgment
**Santos, 2012a [23]**	Low	Some concern	Some concern	Low	Some concern	High
**Santos, 2012b [24]**	Low	Low	Low	Low	Low	Low
**Ashwini, 2015 [32]**	Some concern	High	Low	High	Some concern	High
**Alvarenga, 2017 [33]**	Low	Low	Some concern	Low	Low	Some concern
**Cristine, 2024 [11]**	High	High	Low	High	Some concern	High
**Gondim, 2025 [34]**	Low	Low	Some concern	Low	Low	Some concern

**Table 4:** Risk of bias assessment (ROBINS-I).

ROBINS-I Domain	Bias Due to Confounding	Bias in Selection of Participants into the Study	Bias in Classification of Interventions	Bias Due to Deviations from Intended Interventions	Bias Due to Missing Data	Bias in Measurement of Outcomes	Bias in Selection of the Reported Result	Overall Risk of Bias
**Al-Ashaikh, 2011 [25]**	Serious	Moderate	Low	Moderate	Low–Moderate	Serious	Moderate	Serious risk of bias
**Salman, 2013 [22]**	Serious	Moderate	Low	Moderate	Low–Moderate	Serious	Moderate	Serious risk of bias

**Table 5:** Quality of evidence (GRADE).

Outcome Measured	N. of Part. (Studies)	Study Limitation	Inconsistency	Indirectness	Imprecision	Publication Bias	Overall Quality of Evidence	Effect Estimate MD/SMD [95% CI]	Effect Size
**Pain**	434 (6)	Serious ^a^	Serious ^b^	Not Serious ^c^	Serious ^d^	Possible ^e^	Ꚛꓳꓳꓳ Very Low	SMD, 0.04 [−0.60, 0.68]	Low
**Wound**	282 (5)	Serious ^a^	Serious ^b^	Not Serious ^c^	Serious ^d^	Possible ^e^	Ꚛꓳꓳꓳ Very Low	MD, 0.94 [−0.69, 2.56]	high

GRADE, Grading of Recommendations Assessment, Development and Evaluation; MD: mean difference, SMD: standardized mean difference; CI, confidence interval. ^a^ Study limitation: allocation concealment was not clearly reported, lack of blinding of participants or assessors and therapists, ^b^ Inconsistency: significant heterogeneity in meta-analysis, I^2^ > 75%, ^c^ Indirectness: not serious; patient populations and outcomes aligned with the research question, ^d^ Imprecision: small sample size with wide confidence interval, less than 400 participants, pooled effect crossed the minimally important difference (MID), ^e^: Publication bias: funnel plots were underpowered (<10 studies). Potential small-study effects considered but not formally downgraded, Ꚛꓳꓳꓳ: Very low quality of evidence: The true effect is probably markedly different from the estimated effect with very little confidence in the effect estimate.

## Data Availability

The original contributions presented in this study are included in the article/Supplementary Material. Further inquiries can be directed to the corresponding author.

## Supplementary Materials

The following supporting information can be downloaded at: https://www.mdpi.com/article/10.3390/jcm15030964/s1, File S1: PRISMA 2020 checklist, File S2: PubMed Search Strategy.

## Abbreviations

The following abbreviations are used in this manuscript:

PBM	Photobiomodulation
LLLT	Low-Level Laser Therapy
RCT	Randomized Controlled Trial
VAS	Visual Analog Scale
NRS/NPRS	Numeric Rating Scale/Numeric Pain Rating Scale
REEDA	Redness, Edema, Ecchymosis, Discharge, Approximation scale
NSAIDs	Non-Steroidal Anti-Inflammatory Drugs
SF-MPQ	Short-Form McGill Pain Questionnaire
LED	Light-Emitting Diode
GRADE	Grading of Recommendations Assessment, Development and Evaluation
RoB2	Risk of Bias assessment for randomized controlled trials
ROBINS-I	Risk Of Bias in Non-randomized Studies

## References

[1] Thakar R., Sultan A.H. (2009). Episiotomy and Obstetric Perineal Trauma.

[2] Thacker S.B., Banta H.D. (1983). Benefits and risks of episiotomy: An interpretive review of the English language literature, 1860–1980. Obstet. Gynecol. Surv..

[3] Carroli G., Belizan J. (2000). Episiotomy for vaginal birth. Cochrane Database Syst. Rev..

[4] Jiang H., Qian X., Carroli G., Garner P. (2017). Selective versus routine use of episiotomy for vaginal birth. Cochrane Database Syst. Rev..

[5] Macarthur A.J., Macarthur C. (2004). Incidence, severity, and determinants of perineal pain after vaginal delivery. Am. J. Obstet. Gynecol..

[6] Andrews V., Thakar R., Sultan A.H., Jones P.W. (2008). Occurrence of pain after childbirth and its association with obstetric and maternal factors. BJOG.

[7] Souza J.P., Gulmezoglu A.M., Vogel J., Carroli G., Lumbiganon P., Qureshi Z., Costa M.J., Fawole B., Mugerwa Y., Nafiou I. (2013). Moving beyond essential interventions for reduction of maternal mortality: The WHO Multicountry Survey. Lancet.

[8] World Health Organization (2018). WHO Recommendations: Intrapartum Care for a Positive Childbirth Experience.

[9] Kettle C., Tohill S. (2011). Perineal care. BMJ Clin. Evid..

[10] Beleza A.C.S., Ferreira C.H.J., Driusso P., Dos Santos C.B., Nakano A.M.S. (2017). Effect of cryotherapy on relief of perineal pain after vaginal childbirth with episiotomy: A randomized and controlled clinical trial. Physiotherapy.

[11] Cristine Boniatti Constant É., Plentz Stein G., Camargo de Oliveira K., Laureano Paiva L., Martins Costa S., Geraldo Lopes Ramos J. (2024). Comparison of photobiomodulation with cryotherapy in the immediate postpartum period of parturients with grade I, grade II lacerations and/or episiotomy in reducing perineal and vulvar and edema: A randomized clinical trial. Eur. J. Obstet. Gynecol. Reprod. Biol..

[12] East C.E., Begg L., Henshall N.E., Marchant P.R., Wallace K. (2012). Local cooling for relieving pain from perineal trauma sustained during childbirth. Cochrane Database Syst. Rev..

[13] Steen M., Cooper K., Marchant P., Griffiths-Jones M., Walker J. (2000). A randomized controlled trial comparing cooling treatments for postnatal perineal trauma. Midwifery.

[14] Hamblin M.R. (2017). Photobiomodulation or low-level laser therapy. J. Biophotonics.

[15] Karu T.I. (2003). Low-power laser therapy. Biomedical Photonics Handbook.

[16] Hamblin M.R., Demidova T.N. (2006). Mechanisms of low level light therapy. Proc. SPIE.

[17] Huang Y.-Y., Chen A.C.H., Carroll J.D., Hamblin M.R. (2009). Biphasic dose response in low level light therapy. Dose-Response.

[18] Enwemeka C.S., Parker J.C., Dowdy D.S., Harkness E.E., Sanford L.E., Woodruff L.D. (2004). The efficacy of low-power lasers in tissue repair and pain control. Photomed. Laser Surg..

[19] Hopkins J.T., McLoda T.A., Seegmiller J.G., Baxter G.D. (2004). Low-level laser therapy facilitates superficial wound healing in humans. J. Athl. Train..

[20] Bjordal J.M., Johnson M.I., Iversen V., Aimbire F., Lopes-Martins R.A.B. (2006). Low-level laser therapy in acute pain. Photomed. Laser Surg..

[21] Taha N., Daoud H., Malik T., Shettysowkoor J., Rahman S. (2024). The Effects of Low-Level Laser Therapy on Wound Healing and Pain Management in Skin Wounds: A Systematic Review and Meta-Analysis. Cureus.

[22] Salman S.S., Mohammed K.A. (2013). Enhancement of Episiotomy Healing Using (790–805) nm Diode Laser as a Supplementary Treatment. Iraqi J. Laser.

[23] Santos Jde O., de Oliveira S.M., da Silva F.M., Nobre M.R., Osava R.H., Riesco M.L. (2012). Low-level laser therapy for pain relief after episiotomy: A double-blind randomised clinical trial. J. Clin. Nurs..

[24] Santos Jde O., Oliveira S.M., Nobre M.R., Aranha A.C., Alvarenga M.B. (2012). A randomised clinical trial of the effect of low-level laser therapy for perineal pain and healing after episiotomy: A pilot study. Midwifery.

[25] Al-ashaikh S.F. (2011). Evaluation of Low Level Laser Therapy Using Diode Laser in Enhancement of Episiotomy Wound Healing. Iraqi J. Laser.

[26] Page M.J., McKenzie J.E., Bossuyt P.M., Boutron I., Hoffmann T.C., Mulrow C.D., Shamseer L., Tetzlaff J.M., Akl E.A., Brennan S.E. (2021). The PRISMA 2020 statement: An updated guideline for reporting systematic reviews. BMJ (Clin. Res. Ed.).

[27] Higgins J.P., Altman D.G., Gøtzsche P.C., Jüni P., Moher D., Oxman A.D., Savović J., Schulz K.F., Weeks L., Sterne J.A.C. (2011). The Cochrane Collaboration’s tool for assessing risk of bias in randomised trials. BMJ (Clin. Res. Ed.).

[28] Sterne J.A.C., Savović J., Page M.J., Elbers R.G., Blencowe N.S., Boutron I., Cates C.J., Cheng H.Y., Corbett M.S., Eldridge S.M. (2019). RoB 2: A revised tool for assessing risk of bias in randomised trials. BMJ (Clin. Res. Ed.).

[29] Guyatt G., Oxman A.D., Akl E.A., Kunz R., Vist G., Brozek J., Norris S., Falck-Ytter Y., Glasziou P., DeBeer H. (2011). GRADE guidelines: 1. Introduction-GRADE evidence profiles and summary of findings tables. J. Clin. Epidemiol..

[30] Balshem H., Helfand M., Schünemann H.J., Oxman A.D., Kunz R., Brozek J., Vist G.E., Falck-Ytter Y., Meerpohl J., Norris S. (2011). GRADE guidelines: 3. Rating the quality of evidence. J. Clin. Epidemiol..

[31] Kymplová J., Navrátil L., Knízek J. (2003). Contribution of phototherapy to the treatment of episiotomies. J. Clin. Laser Med. Surg..

[32] Chougala A., Mahishale A. (2015). A Randomized Clinical Trial to evaluate the Effect of Therapeutic Ultrasound and Low-level Laser Therapy on Perineal Pain following Vaginal Delivery with Episiotomy. J. S. Asian Fed. Obstet. Gynaecol..

[33] Alvarenga M.B., de Oliveira S.M., Francisco A.A., da Silva F.M., Sousa M., Nobre M.R. (2017). Effect of low-level laser therapy on pain and perineal healing after episiotomy: A triple-blind randomized controlled trial. Lasers Surg. Med..

[34] Gondim E.J.L., Nascimento S.L., Gaitero M.V.C., Mira T.A.A., Gonçalves A.V., Surita F.G. (2025). Effects from a single application of photobiomodulation on pain intensity from perineal trauma related to childbirth: A randomized controlled trial. Int. J. Gynaecol. Obstet..

[35] Zhao H., Hu J., Zhao L. (2021). The effect of low-level laser therapy as an adjunct to periodontal surgery in the management of postoperative pain and wound healing: A systematic review and meta-analysis. Lasers Med. Sci..

[36] Mosca R.C., Ong A.A., Albasha O., Bass K., Arany P. (2019). Photobiomodulation Therapy for Wound Care: A Potent, Noninvasive, Photoceutical Approach. Adv. Skin. Wound Care.

[37] Alayat M.S., El-Sodany A.M., Ebid A.A., Shousha T.M., Abdelgalil A.A., Alhasan H., Alshehri M.A. (2018). Efficacy of high intensity laser therapy in the management of foot ulcers: A systematic review. J. Phys. Ther. Sci..

[38] Yoon S.H., Huh B.K., Abdi S., Javed S. (2024). The efficacy of high-intensity laser therapy in wound healing: A narrative review. Lasers Med. Sci..

[39] Thabet A.A.E., Mahran H.G., Ebid A.A., Alshehri M.A. (2018). Effect of pulsed high intensity laser therapy on delayed caesarean section healing in diabetic women. J. Phys. Ther. Sci..

[40] Ebid A.A., El-Kafy E.M., Alayat M.S. (2013). Effect of pulsed Nd:YAG laser in the treatment of neuropathic foot ulcers in children with spina bifida: A randomized controlled study. Photomed. Laser Surg..

[41] Posten W., Wrone D.A., Dover J.S., Arndt K.A., Silapunt S., Alam M. (2005). Low-level laser therapy for wound healing: Mechanism and efficacy. Dermatol. Surg..

[42] Andrade Fdo S., Clark R.M., Ferreira M.L. (2014). Effects of low-level laser therapy on wound healing. Rev. Col. Bras. Cir..

