# Supplementary material for: The Effectiveness of Photobiomodulation Therapy on Perineal Pain and Wound Healing After Episiotomy—A Systematic Review and Meta-Analysis
Source: J Clin Med. 2026 Jan 25;15(3):964. doi: 10.3390/jcm15030964 (PMC12897679; doi:10.3390/jcm15030964)
Supplement: Supplementary file 1 [file jcm-15-00964-s001.zip › Supplementary File S2. PubMed Search Strategy.pdf]

PubMed Search Strategy

Database: PubMed (MEDLINE)

Date of Search: 26 October 2025

Search Objective

To identify studies evaluating the effectiveness of laser therapy or photobiomodulation on pain relief and perineal wound healing in postpartum women following episiotomy or perineal tear repair.

Search Strategy Development

The search strategy was developed using a combination of Medical Subject Headings (MeSH) and free-text keywords, structured according to the PICO framework. Boolean operators (AND, OR) were applied.

Population (Postpartum women after episiotomy or perineal tear)

("Postpartum Period"[Mesh] OR "Puerperium"[Mesh] OR postpartum OR postnatal OR puerperal OR "Episiotomy"[Mesh] OR episiotomy OR "Perineum/injuries"[Mesh] OR "Perineal Lacerations"[Mesh] OR perineal tear OR perineal trauma OR obstetric laceration OR vaginal delivery)

Intervention (Laser therapy / Photo biomodulation)

("Laser Therapy"[Mesh] OR "Phototherapy"[Mesh] OR "Low-Level Light Therapy"[Mesh] OR "Photobiomodulation Therapy"[Mesh] OR laser therapy OR low-level laser OR low intensity laser OR diode laser OR infrared laser OR laser irradiation OR photobiomodulation OR LLLT OR PBM)

Outcomes (Pain relief and wound healing)

("Pain"[Mesh] OR pain OR perineal pain OR pain relief OR "Wound Healing"[Mesh] OR wound healing OR tissue repair OR healing time OR "Inflammation"[Mesh] OR inflammation OR edema OR infection OR scar OR scar tissue)

Final Combined Search Strategy

(("Postpartum Period"[Mesh] OR "Puerperium"[Mesh] OR postpartum OR postnatal OR puerperal OR "Episiotomy"[Mesh] OR episiotomy OR "Perineum/injuries"[Mesh] OR "Perineal Lacerations"[Mesh] OR perineal tear OR perineal trauma OR obstetric laceration OR vaginal delivery)) AND ("Laser Therapy"[Mesh] OR "Phototherapy"[Mesh] OR "Low-Level Light Therapy"[Mesh] OR "Photobiomodulation Therapy"[Mesh] OR laser therapy OR low level laser OR low intensity laser OR diode laser OR infrared laser OR laser irradiation OR photobiomodulation OR LLLT OR PBM) AND ("Pain"[Mesh] OR pain OR
perineal pain OR pain relief OR "Wound Healing"[Mesh] OR wound healing OR tissue repair OR healing time OR "Inflammation"[Mesh] OR inflammation OR edema OR infection OR scar OR scar tissue)

Search Limits

Language: English

Population: Humans

Publication type: Randomized Controlled Trial

Search Results

Records retrieved from PubMed: **17**

-Reference lists of included studies were manually screened to identify additional relevant articles. The search strategy was adapted for other databases.
